# Solid Tumor Microenvironment Can Harbor and Support Functional Properties of Memory T Cells

**DOI:** 10.3389/fimmu.2021.706150

**Published:** 2021-11-11

**Authors:** Peter M. Sullivan, Steven James Reed, Vandana Kalia, Surojit Sarkar

**Affiliations:** ^1^ Ben Towne Center for Childhood Cancer Research, Seattle Children’s Research Institute, Seattle, WA, United States; ^2^ Department of Pediatrics, Division of Hematology and Oncology, University of Washington, Seattle, WA, United States; ^3^ Department of Pathology, University of Washington School of Medicine, Seattle, WA, United States

**Keywords:** bystander memory anti-tumor immunity, CAR T therapy, tumor microenvironment, chemokines, CXCR3, antigen

## Abstract

Robust T cell responses are crucial for effective anti-tumor responses and often dictate patient survival. However, in the context of solid tumors, both endogenous T cell responses and current adoptive T cell therapies are impeded by the immunosuppressive tumor microenvironment (TME). A multitude of inhibitory signals, suppressive immune cells, metabolites, hypoxic conditions and limiting nutrients are believed to render the TME non-conducive to sustaining productive T cell responses. In this study we conducted an in-depth phenotypic and functional comparison of tumor-specific T cells and tumor-nonspecific bystander memory T cells within the same TME. Using two distinct TCR transgenic and solid-tumor models, our data demonstrate that despite exposure to the same cell-extrinsic factors of the TME, the tumor-nonspecific bystander CD8 T cells retain the complete panoply of memory markers, and do not share the same exhaustive phenotype as tumor-reactive T cells. Compared to tumor-specific T cells, bystander memory CD8 T cells in the TME also retain functional effector cytokine production capabilities in response to *ex vivo* cognate antigenic stimulation. Consistent with these results, bystander memory T cells isolated from tumors showed enhanced recall responses to secondary bacterial challenge in a T cell transplant model. Importantly, the tumor-resident bystander memory cells could also efficiently utilize the available resources within the TME to elaborate *in situ* recall effector functions following intra-tumoral peptide antigen injection. Additionally, CRISPR-Cas9 gene deletion studies showed that CXCR3 was critical for the trafficking of both tumor antigen-specific and bystander memory T cells to solid tumors. Collectively, these findings that T cells can persist and retain their functionality in distinct solid tumor environments in the absence of cognate antigenic stimulation, support the notion that persistent antigenic signaling is the central driver of T cell exhaustion within the TME. These studies bear implications for programming more efficacious TCR- and CAR-T cells with augmented therapeutic efficacy and longevity through regulation of antigen and chemokine receptors.

## Introduction

The limited success of adoptive T cell immunotherapy against solid tumors has been attributed to a multitude of variables including the trafficking of infused T cells to solid tumors and subsequent penetration and infiltration into the tumor microenvironment (TME) (1–3). In addition to chronic antigenic signaling, the TME harbors a multitude of inhibitory signals (i.e. PD-L1, IL-10, TGF-β), suppressive immune cells (i.e. regulatory T cells, (T_reg_); monocyte derived suppressor cells, MDSC), metabolites (i.e. kynurenine metabolites), hypoxic conditions and limiting nutrients, which are believed to render the TME non-conducive to sustaining productive T cell responses (4–7). Consequently, most tumor-reactive T cells develop a hallmark exhaustive state characterized by loss of functionality and impaired memory differentiation, thus compromising anti-tumor immunity (2).

Current strategies to prevent T cell exhaustion and prolong T cell function within the TME are largely focused on targeting immune checkpoint molecules such as PD-1/PD-L1 and CTLA4 (8–11). However, only a fraction of patients receiving T cell immunotherapy for solid tumors are responsive to immune checkpoint blockade (ICB) (8), and responsiveness appears to depend on the retention of a stem-cell like phenotype by T cells which is sequentially lost as T cells become terminally exhausted (12–17). Hence, developing alternative strategies to combat T cell exhaustion and dysfunction in the TME will be instrumental in enhancing future adoptive T cell therapies against solid tumors, and expanding the reach of current ICB combination therapies to more patients. To develop such strategies, a greater understanding of the contributions of the individual immunosuppressive TME factors on T cell exhaustion, stemness, and responsiveness to ICB must be established.

Recent studies have identified non-tumor-antigen specific, or bystander T cells, within the TME (18–21). Bystander memory T Cells within the TME appear to retain their functionality, as the activation of bystander memory T cells within the TME has been shown to enhance the general anti-tumor response (18, 19) by inducing a local pro-inflammatory environment and production of effector cytokines such as IL-2 (22). Collectively, these findings suggest that T cell dysfunction in the TME is not a result of immunosuppressive factors alone but occurs in combination with chronic antigenic stimulation.

Multiple types of tumors have been shown to harbor bystander cells even when tumor antigen-specific cells are not detectable (20, 23, 24). These intriguing observations raise the question whether bystander memory T cells display superior trafficking to solid tumors compared to naïve tumor-specific T cells. Determining the mechanisms behind the migration of bystander memory T cells to solid tumors may guide immunotherapeutic approaches for both tumor-reactive T cells and harnessing the potential of bystander memory T cell activation in the TME to augment the anti-tumor response.

Here, we focus on ascertaining the in-depth phenotype, function, and memory recall potential of bystander memory T cells by comparing tumor-specific T cells and tumor-nonspecific bystander memory T cells within the same TME, using two distinct solid tumor models. We demonstrate that while tumor-specific T cells developed a characteristic exhaustive state within the TME (4–7), bystander memory T cells in the same tumors retained their expression of markers associated with canonical memory T cells … Further studies on T cell functionality showed that bystander memory T cells isolated from solid tumors retained their capacity for rapid effector cytokine production upon restimulation both *ex vivo* and *in situ*, and generated canonical recall responses to viral infection. Similar to reports of antigen-specific T cells migration to solid tumors (25, 26), the trafficking of bystander memory T cells to solid tumors was found to be largely dependent on CXCR3. Finally, we extend our findings to show that tumor-resident bystander memory T cells show similar resistance to exhaustion in a murine model of CAR T cell immunotherapy.

Collectively, the results from this study reveal a mechanism for antigen-independent trafficking of T cells to solid tumors, and directly demonstrate the impact of antigenic signaling in driving T cell exhaustion within the TME. These findings highlight the potential for bioengineering strategies to enhance adoptive T cell therapy against solid tumors *via* increased T cell migration to solid tumors through chemokine receptor engineering (27), and combatting T cell exhaustion through tunable antigen receptor expression (28–34). Additionally, these studies support potential targeting of memory bystander T cells to augment the PD-1 checkpoint blockade responsiveness of adoptively transferred CAR T cells, as in the case of TCR transgenic T cell therapies (18–21).

## Methods and Materials

### Animals

C57BL/6 mice were purchased from Jackson Laboratory (Bar Harbor, ME, USA). Ly5.1^+^ H-2K^b^ Ovalbumin-specific TCR transgenic OT-I mice were provided by Dr. Martin Prlic (Fred Hutchinson Cancer Resource Center). Thy1.1^+^ H-2D^b^ GP33-specific TCR-transgenic P14 mice were maintained in our colony. *Listeria monocytogenes* expressing the ovalbumin peptide (Lm-Ova) was used at 1x10^5^ CFU and injected intravenously and LCMV_Arm_ was used at 2x10^5^ PFU and injected intraperitoneally. All procedures were approved by IACUC and conducted in accordance to institute guidelines.

### Flow Cytometry

All antibodies were purchased from Biolegend (San Diego, CA, USA). Aqua fluorescent reactive dye was purchased from Invitrogen. 2x10^6^ cells were stained for surface or intracellular proteins by incubating cells with antibodies for 45 minutes on ice, fixed and permeabilized with 1x Cytofix/CytoPerm (BD Biosciences), then stained for 45 minutes for intracellular proteins with antibodies diluted in 1x Permwash, before being fixed in 2% PFA for 20 minutes as described previously (35–39). All samples were acquired on a LSRII Fortessa (BD Biosciences, San Jose, CA, USA) and analyzed with FlowJo V9 software.

### Isolation, Adoptive Transfers, and Sorting of CD8 T Cells

CD8 T cells were isolated from spleens using MojoSort Mouse CD8 T Cell Isolation Kit (Biolegend). CD8 T cells were adoptively transferred intravenously at the indicated numbers. OT-I bystander memory cells were sorted on a FACSJazz (BD Biosciences) using antibodies specific to Ly5.1.

### Intracellular Cytokine Staining

About 2x10^6^ lymphocytes were stimulated with 0.2 µg/ml GP33-41 peptide, 0.2μg/ml Ovalbumin peptide, or plate-coated αCD3/αCD28 for 5 hours in the presence of Brefeldin A (BFA), followed by surface staining and intracellular staining for IFN-γ, TNFα, and IL-2.

### Intratumoral Cytokine Production

BFA, Ovalbumin peptide, and GP33-41 peptide in a total volume of 30 μl was injected directly into tumors. After 5 hours, the spleen and tumor were harvested and lymphocytes isolated. 2x10^6^ cells from each tissue were stained as described above.

### Tumor Cells

MC38 and B16.F10 cell lines were obtained from ATCC. These lines were transduced with lentivirus to express EGFP, firefly luciferase, and the LCMV GP33-41 antigen. The lines were clonally selected and expanded. For tumor assays, 1x10^6^ tumor cells were injected subcutaneously on the right flank of the mouse. Tumor measurements began 7 days post tumor cell injection and were carried out every 2-3 days afterward. Tumor volume was calculated as length*(width^2^)/2.

### CRISPR/Cas9

The CXCR3 gene was edited for deletion using CRISPR/Cas9 with three guide RNAs targeting the CXCR3 gene simultaneously. Guide RNAs were designed and ordered from Integrated DNA Technologies (IDT). The RNA sequences used were 1. TCTGCGTGTACTGCAGCTAG, 2. TGAGGGCTACACGTACCCGG, and 3. AGTTAACACCAGCAGAACAT. The RNP complex was produced using Alt-R s.p. Cas9 Nuclease V3 protein (IDT), Alt-R CRISPR-Cas9 tracrRNA with ATTO550 (IDT), and Alt-R CRISPR-Cas9 sgRNA targeted to CXCR3 (IDT). The RNP complex was introduced using the Neon Transfection System (ThermoFisher Scientific). Uptake of the RNP complex was verified by ATTO550 staining using flow cytometry and CXCR3 knockout was confirmed by antibody staining and flow cytometry.

### CAR T Cell Design and Transduction

An αCD19 CAR based on published methods was constructed in a MP71 vector (Chen et al, 2019). Retrovirus was produced by transient transfection of Plat E cells (Cell Bio Labs). CD8 T cells were isolated using the MojoSort Mouse CD8 T Cell Isolation kit (Biolegend). Cells were activated by plate bound αCD3/αCD28 for 24 hours then spinoculated by centrifuging at 2000x*g* for 60 minutes at 32°C. Cells were then adoptively transferred into day 1 LCMV_Arm_ infection matched mice.

### Statistical Analysis

Paired or unpaired Student’s t-tests as appropriate were used to evaluate differences between samples. ANOVA with multiple comparisons was used to evaluate statistical significance between three or more groups. All analysis was performed using Graphpad Prism. P values of statistical significance are indicated with an asterisk: *p<0.05, **p<0.01, ***p<0.001. p>0.05 were considered non-significant (ns).

## Results

### Bystander Memory CD8 T Cells Infiltrate Established Solid Tumors

**Figure 1:**
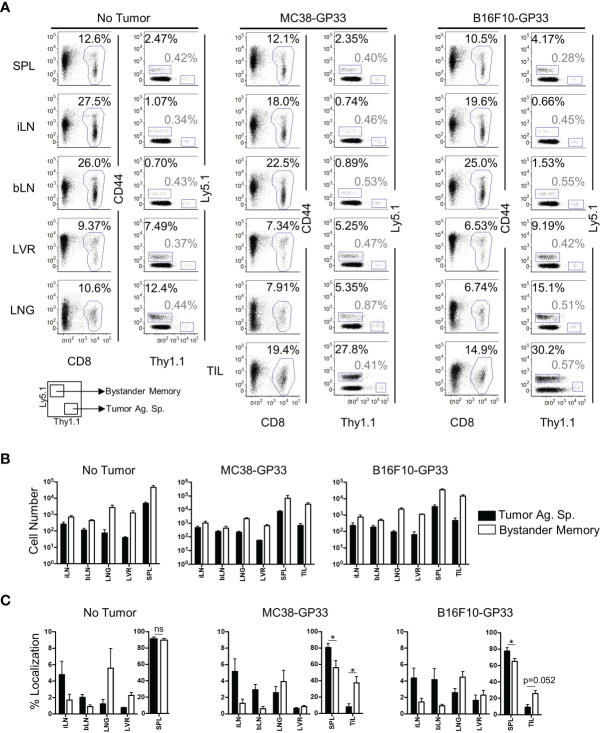
Bystander Memory T Cells infiltrate into established solid tumors. WT OT-I CD8 T cells were adoptively transferred into C57BL6 mice and infected with LM-Ova. Following memory differentiation (>day 30 post infection), naïve P14 CD8 T cells were transferred into the mice. The mice were then subcutaneously injected with MC38-GP33 or B16.F10-GP33 tumor cells. **(A)** FACS plots of CD8 T cells in spleen (SPL), brachial lymph node (bLN), inguinal lymph node, (iLN), liver (LVR), lung (LNG), and tumor infiltrating lymphocytes (TIL) show the frequency of donor CD8 T cells of total CD8 T cells or the frequency of bystander memory OT-I donors (black) and tumor antigen specific P14 donors (gray) of total CD8 T cells at day 21 post tumor injection. Bar graphs show **(B)** the total number of CD8 T cells, bystander memory, and tumor antigen specific T cells in each tissue and **(C)** the percent localization of bystander memory cells and tumor antigen specific CD8 T cells in each tissue. Percent localization was calculated as total number of specified cell population in a given tissue divided by sum of that cell population identified in all the tissues collected. Representative plots are shown from N=5 mice. Significance was determined by paired T-test. *p < 005. Differences were non-significant if not otherwise indicated. Data is representative of 3 separate experiments. ns, non-significant.

Solid tumors have been recently reported to harbor bystander memory T cells (18–20), however the origin of these cells has not been studied extensively. To examine the role of antigen-specificity in CD8 T cell trafficking to the TME, we compared the ability of bystander memory T cells and tumor-antigen-specific (tumor-specific) T cells to traffic to tumors following adoptive co-transfer into mice. Briefly, naïve OT-1 T cells were transferred into naïve C57Bl/6 mice which were then infected with LM-Ova to generate Ova-specific OT-I (bystander) memory CD8 T cells (**Figure S1A**). About 30 days after infection, naive TCR transgenic P14 CD8 T cells specific for the LCMV GP33 epitope were transferred into the OT-1 memory mice, which were subsequently inoculated with GP33-expressing MC38 colon carcinoma or B16.F10 melanoma tumors (**Figure S1A**). By 21 days post-tumor inoculation, the tumors were well established (**Figure S1B**) and both the bystander memory and tumor-specific donor cells were detectable in the spleen, inguinal (tumor-proximal) and brachial (tumor-distal) lymph nodes, liver, lung, and tumor sites (**Figures 1A, B** and **S1C**). In the non-tumor bearing mice, bystander and tumor-specific populations of cells showed largely similar distribution patterns across the various tissues (**Figure 1B, C**). However, in tumor-bearing mice, the tumor-specific CD8 T cells were redistributed from spleens to the tumor sites as indicated by decrease in absolute donor cell numbers, as well as percent localization when compared to non-tumor bearing control mice (**Figures 1B, C** and **S1D**). These results suggest that, while T cells may traffic to solid tumors from all tissues examined, the spleen acts as the primary reservoir for cells recruited to solid tumors (**Figures 1C** and **S1D**). Somewhat unexpectedly, a significantly greater proportion of the bystander memory cell population was found in both the MC38 and B16.F10 tumors compared to the naïve tumor-specific cells (**Figures 1C** and **S1D**). These data demonstrate that bystander memory cells are effectively recruited to solid tumors in an antigen-independent manner.

### Bystander Memory CD8 T Cells Maintain a Quiescent Phenotype in the TME

**Figure 2:**
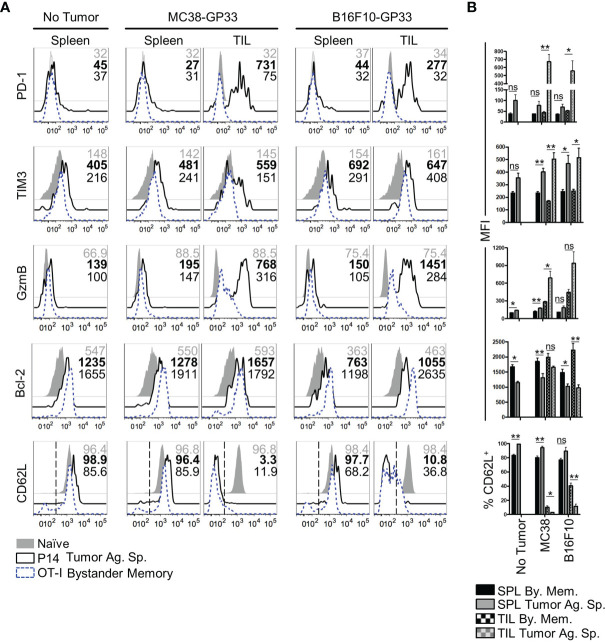
Phenotype of bystander memory CD8 T cells in tumor microenvironment. **(A)** Histograms are gated on CD8 T cells and show the respective markers in spleen of naïve (gray), tumor antigen specific (solid, black), or bystander memory (dashed, blue) CD8 T cells. Numbers show MFI of given markers for naïve (gray), tumor antigen specific (bold), and bystander memory (black) T cells for spleen and TIL taken from B6 mice with no tumor, MC38-GP33 tumor, or B16.F10-GP33 tumor. Bar charts to the right **(B)** show the average MFI or average percent positive with SEM for each population of CD8 T cells. Representative plots are shown from N=5 mice per group. *p < 0.05. **p < 0.01 as determine by paired T-test. Data is representative of 3 independent experiments. ns, non-significant.

The rapid exhaustion of tumor-Ag sp. T cells in the TME has been attributed to chronic antigenic stimulation in combination with a multitude of cell extrinsic variables. Such factors include inhibitory receptor ligands and cytokines found on immunosuppressive cells in the TME and tumor cells, nutrient deprivation, and a hypoxic microenvironment (4, 5, 40, 41). In comparison to how these factors influence responding T cells, even less is known about their influence on T cell programming and function in the absence of antigenic signaling. To independently evaluate roles of antigenic signaling *vs* cell-extrinsic variables on CD8 T cell exhaustion in the TME, we compared the phenotype of bystander memory and tumor-specific T cells isolated from tumors and spleens of mice, as in **Figure 1**. Consistent with T cell phenotypes in an antigen-free environment, both the bystander memory and tumor-specific cells isolated from the spleens of naïve mice showed a quiescent phenotype as elucidated by low levels of expression of GzmB, the exhaustion markers PD-1 and TIM-3, and high expression levels of the pro-survival marker Bcl-2 and lymph node homing marker L-selectin (CD62L) (**Figure 2A**). Bystander and tumor-specific T cells isolated from the spleens of tumor bearing mice displayed a similar phenotype to those from the non-tumor bearing controls, suggesting that negligible amounts of tumor-Ag were present in the spleens of tumor-bearing mice (**Figure 2A**). In contrast, striking phenotypic differences between bystander and tumor-specific T cells were observed in tumor infiltrating lymphocytes (TIL) isolated from both MC38 and B16.F10 tumors. While the tumor-specific T cells isolated from the tumors displayed a phenotype characteristic of strong antigenic signaling and possible exhaustion, the bystander memory cells largely retained a phenotype similar to those isolated from the spleen (**Figure 2A**). Compared to bystander memory T cells, the tumor-specific TIL exhibited significantly higher expression levels of GzmB, PD-1, TIM-3, and significantly lower expression levels of Bcl-2, and significantly reduced proportions of CD62L^+^ cells (**Figure 2B**). The bystander memory TIL population also contained a significantly lower frequency of CD38 and CD101 double-positive cells compared to the tumor-specific T cells, thus indicating that bystander memory cells in the TME are resistant to terminal differentiation. (**Figure S2A**). Hence, despite exposure to the harsh and immunosuppressive cell extrinsic variables in the TME, the bystander cells evidently retained a quiescent, and largely undifferentiated state. These results strongly support the notion that chronic antigenic signaling is the predominant factor driving an exhausted phenotype in the tumor-specific T cells within the TME, and mere exposure to environmental factors in the TME does not result in T cell exhaustion.

### Bystander Memory T Cells Maintain Functionality in the Tumor Microenvironment

**Figure 3:**
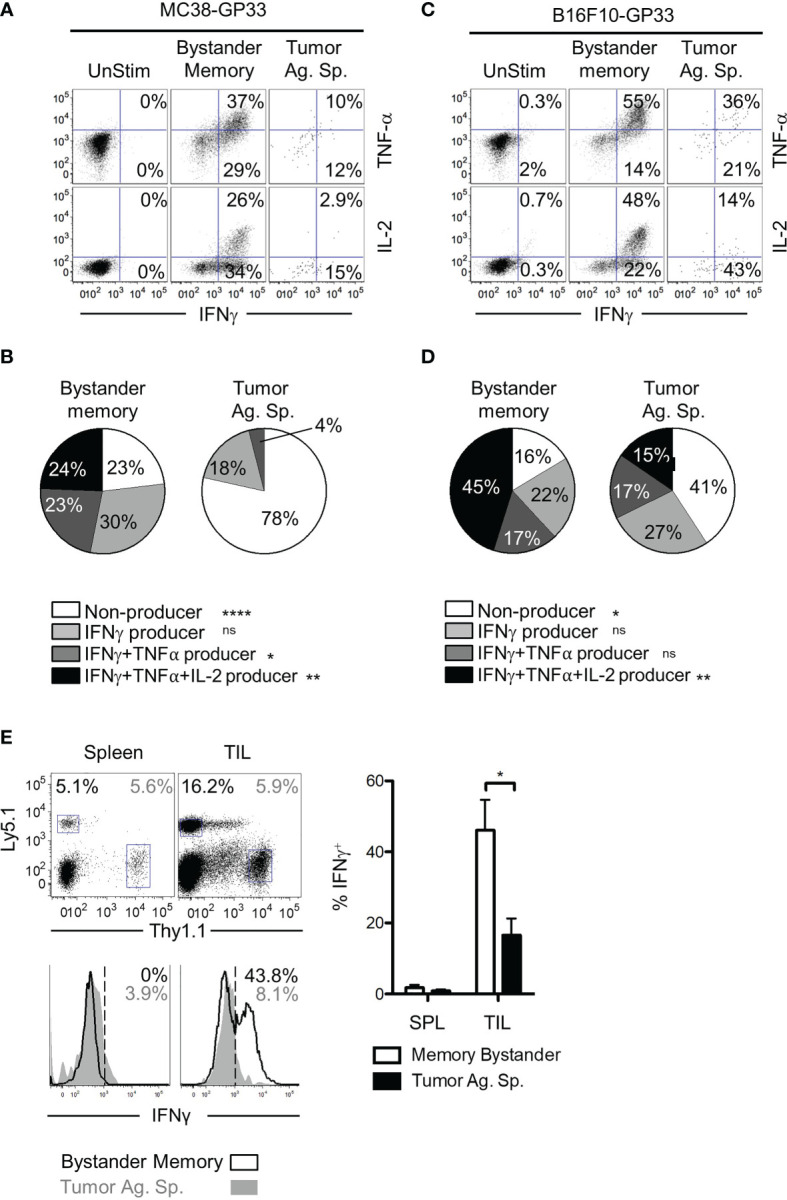
Polyfunctionality of bystander memory T cells in tumor microenvironment. CD8 T cells from spleen and tumor of mice bearing MC38-GP33 tumors **(A, B)** or B16.F10-GP33 tumors **(C, D)** were stimulated with αCD3/αCD28 for 5 hours in the presence of BFA and cytokine production was assessed. **(A, C)**. FACS plots are gated on total donor population (unstim), OT-I donors (bystander memory) or P14 donors (tumor ag-sp) cells from tumors. FACS plots show % of IFN-γ/TNF-α double positive or % IFN-γ/IL-2 double positive of each population. **(B, D)**. To assess the degree of polyfunctionality of each population of cells, the proportion of nonproducing (white), IFN-γ^+^ (light gray), IFN-γ^+^ TNFα^+^ (dark gray) and IFN-γ^+^ TNFα^+^ IL-2^+^ (black) donor CD8 T cells were plotted in pie charts. **(E)**. Established B16.F10-GP33 tumors were injected intratumorally with 30 ul PBS containing Ova peptide, GP33 peptide, and BFA to assess *in situ* cytokine response. Five hours later, the spleen and tumors were collected, and cells were assessed for IFN-γ production. Gating of donor population is shown in the top panel, with Ly5.1^+^ memory bystanders and Thy1.1^+^ tumor antigen specific CD8 T cells. Bottom panel show histograms for IFN-γ in the spleen (left) and tumor (right). Percentage of IFN-γ positive cells is shown in the upper right corner and is plotted in the bar chart to the right. Representative plots are shown from N=5 mice per group. Statistical significance was determined by ANOVA with multiple comparisons **(B, D)** or paired t-test **(E)** *p < 0.05, Data is representative of 3 independent experiments. **p < 0.01, ****p < 0.0001, ns, non-significant.

T cell dysfunction in the TME can result from T cell exhaustion, anergy, or senescence (42, 43). While induction of inhibitory receptors (such as PD-1 and TIM-3) is a key phenotype of exhausted CD8 T cells, PD-1 is also induced during early stages of activation in acute infections (44). Therefore, we next confirmed whether the bystander memory TILs retained their functionality in the TME, consistent with their lack of an exhausted phenotype. To assess the functionality of bystander memory and tumor-specific T cells, we first evaluated the cytokine production of each population following direct *ex vivo* stimulation with plate-bound αCD3/αCD28. Consistent with the phenotypes observed in cells recovered from spleen and TILs in **Figure 2**, the bystander memory T cells from both spleen and tumor showed strong cytokine production following restimulation (**Figures S3A**). Of the cells isolated from MC38 tumors, the bystander memory cells showed superior cytokine production to the tumor-specific T cells and contained on average 3.7-fold more IFN-γ^+^ TNFα^+^ cells, and nearly 9-fold more IFN-γ^+^ IL-2^+^ cells than tumor-specific T cells (**Figures 3A, B**). Similar patterns were observed in TILs from B16.F10 tumors, where the bystander cells contained 1.5-fold more IFN-γ^+^ TNFα^+^, and 3.4-fold more IFN-γ^+^ IL-2^+^ cells compared to tumor-Ag sp. T cells (**Figures 3C, D** **)**. To further evaluate the functional *vs* exhaustive phenotype of the bystander memory T cells, we compared the polyfunctionality of the bystander memory and tumor-specific T cells following stimulation. Consistent with the exhausted phenotype displayed by the tumor-specific TILs (**Figures 2A** and **S3D, E**), IFN-γ^+^ TNFα^+^ IL-2^+^ tumor-specific T cells were undetectable in MC38 tumors, and on average made up only 15% of tumor-specific T cells isolated from B16.F10 tumors. In contrast, the bystander memory population isolated from the MC38 and B16.F10 tumors contained an average of 24% and 45% of IFN-γ^+^ TNFα^+^ IL-2^+^ T cells, respectively, following restimulation (**Figures 3B, D** **)**. These results are consistent with the phenotypes observed in **Figure 2**, and indicate that despite residency in the distinct TME, bystander memory T cells retain functionality.

The results from the *ex vivo* restimulation demonstrate that compared to tumor-specific CD8 T cells, the bystander memory T cells retain their polyfunctionality following exposure to the TME. However, *in vitro* conditions do not recapitulate the immunosuppressive environment of the TME. To test whether the bystander memory T cells were capable of elaborating effector cytokine production within the TME, T cells in B16.F10-GP33 tumors were directly restimulated *in vivo* through intratumoral injection of GP33 and OVA peptides, and Brefeldin A (BFA). Five hours after peptide injection, tumor-Ag sp. and bystander memory T cells were isolated from tumors and spleens, then immediately examined for intracellular IFN-γ production (**Figure S3F**). As expected, neither the tumor-specific nor bystander memory T cells isolated from the spleens expressed IFN-γ, due to the localized administration of peptide-Ag inside the tumors (**Figure 3E**). Meanwhile, amongst the TILs, there were significantly more IFN-γ-producing bystander memory T cells (44% avg) compared to the tumor-specific T cells (17.5% avg) (**Figure 3E**). The IFN-γ expression patterns observed in the TILs were reflective of their exhausted state as determined by PD-1 expression (**Figure S3G**). In addition, a greater frequency of the bystander memory cells with intermediate and high levels of PD-1 expression were IFN-γ^+^ compared to tumor-specific counterparts (**Figure S3G**). Collectively, using both *ex vivo* and *in situ* stimulation with cognate peptide antigens, these studies confirm that bystander memory T cells in solid tumors maintain their functionality as exemplified by their rapid and robust cytokine production, even within the immunosuppressive TME.

### Memory Bystander T Cells Retain Their Recall Potential Following Residency in the TME

**Figure 4:**
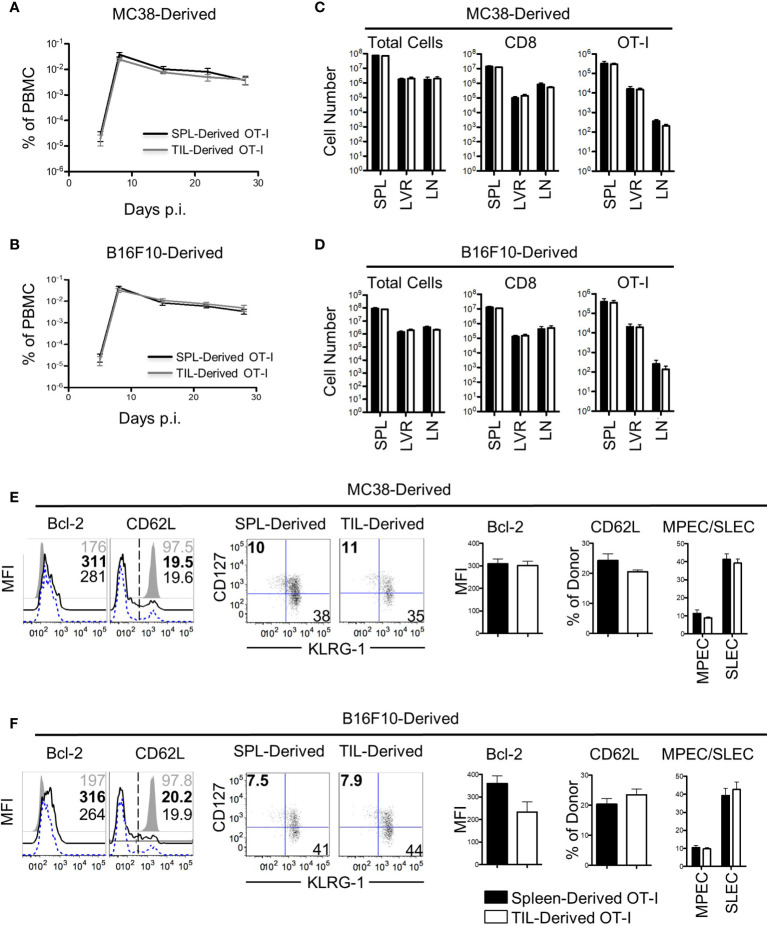
Bystander memory CD8 T cells from the tumor microenvironment retain their recall ability. Bystander memory CD8 T cells were isolated from TILs or spleens of mice bearing MC38-GP33 tumors and were sorted by FACS. 4000 OT-I bystander memory cells were adoptively transferred in naïve B6 mice and subsequently infected with 15k CFU of Lm-Ova. **(A, C)**. Mice were bled to follow donor expansion and contraction. Spleen (SPL)-derived (black) and TIL-derived (gray) bystander memory cells are plotted as percent of total PBMC. Bystander memory isolated from MC38-GP33 bearing mice are shown in **(A)** and bystander memory from B16.F10-GP33 bearing mice in **(C, D)**. At day 28, tissues were collected from these mice and donor cells from SPL, liver (LVR) and lymph node (LN) were analyzed. The total number of cells, total number of CD8 T cells, and OT-I donor cells are quantified from spleen-derived donors (black bars) and tumor-derived donors (white bars). Bystander memory isolated from MC38-GP33 bearing mice are shown in **(B)** and bystander memory from B16.F10-Gp33 bearing mice in **(D, E)** Spleens samples of mice that received donors originating from MC38-GP33 bearing mice **(E)** or from B16.F10-GP33 **(F)** were stained for phenotypic markers. Histograms depict the mean fluorescence intensity (MFI) or percent of cells expressing the given marker. Quantification of each marker is shown to the right. Short-live effector cells (SLEC) were gated on KLRG1+CD127- populations and memory precursor effector cells (MPEC) were gated on KLRG1-CD127+ populations. Unpaired T-tests were run to compare the SPL-derived donors to the TIL-derived donors with no significant differences found between any groups. N=5 mice per group. Data is representative of 2 independent experiments.

The results thus far demonstrated that the cell-extrinsic variables in the TME had little effect on bystander memory exhaustion and polyfunctionality. We next investigated the impact of immunosuppressive factors within the TME on bystander memory T cells’ recall expansion potential – another hallmark functional property of robust memory CD8 T cells. To test this, OT-I bystander memory T cells were isolated from the spleens and tumors of B16.F10-GP33 and MC38-GP33 bearing mice 21 days after tumor inoculation, (**Figure S4A**). Equal numbers of bystander memory CD8 T cells from tumors and spleens were then transferred separately into naive B6 mice which were subsequently challenged with LM-Ova. T cell expansion kinetics were used to evaluate memory responses. Nearly identical expansion and contraction dynamics were observed between OT-I donors originating from the spleen and tumors of both MC38 and B16.F10 recipients (**Figures 4A, B** **)**. Analysis of the spleen, liver, and lymph nodes on D28 post-infection (P.I.) showed similar OT-I cell numbers between spleen and tumor-derived donors (**Figures 4C, D** **)**. Phenotypically, the spleen and tumor-derived donors expressed similar levels of Bcl-2, TIM3, CXCR3, PD-1, and had undergone similar patterns for memory *vs* effector differentiation as determined by CD62L, CD127, and KLRG-1 (**Figures 4E, F**, and **S4B, C**). Furthermore, the spleen and tumor-derived donors were equally functional when stimulated *ex vivo* with αCD3/αCD28 or with Ova peptide (**Figures S4D, E**). Collectively, these data demonstrate that the residency of bystander memory T cells in the TME does not impair CD8 T cell memory differentiation with respect to recall expansion potential and are consistent with our data showing retention of polyfunctionality of bystander memory T cells in tumor and secondary lymphoid sites alike.

### CXCR3 Is Critical for Bystander Cell Localization to Solid Tumors

**Figure 5:**
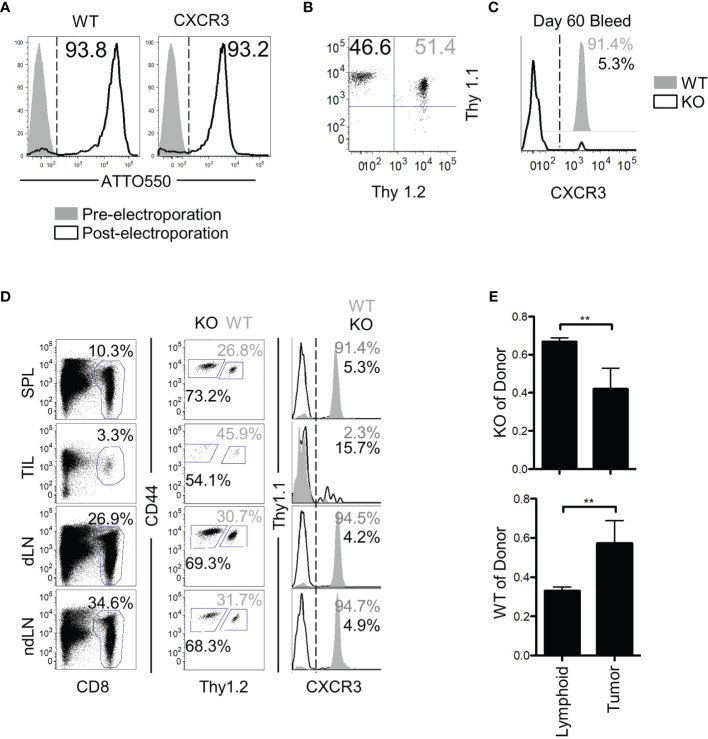
CXCR3 is necessary for proper localization of bystander memory to the tumor. **(A)** Histograms of CD8 T cells pre- and post-Neon electroporation to show percent of cells that acquired ATTO550-labeled CRISPR-Cas9 RNP complex. Percent WT of donor is shown in solid grey, KO in black. **(B)** Cells were mixed 1:1 before adoptive transfer into B6 mice. **(C)** Confirmation of loss of CXCR3 protein in the knockout population at memory. **(D)** Representative plots of total CD8 and of donor populations in each tissue collected. Percent of CD8 is shown above the CD8 gate. Percent of *cxcr3* WT (gray) and KO (black) out of total donor CD8 cells is shown adjacent to each gate (middle column). Confirmation of *cxcr3* KO in each tissue is shown by histogram for CXCR3 expression. Percent WT of donor is shown in solid grey, KO in black. **(E)** Bar graphs showing the proportion of *cxcr3* KO (top) or WT (bottom) CD8 T cells to total donor cells present in either lymphoid tissues (Spleen and Lymph Nodes combined) or in tumors. **p < 0.01 as determined by paired T-test. Representative plots are shown from N=5 mice per group. Data is representative of 2 independent experiments.

Despite the lack of an antigenic target, the bystander memory cells displayed efficient trafficking to solid tumors (**Figure 1**). While the chemokine receptor CXCR3 has been shown to be critical for tumor-specific T cell migration to solid tumors, it is unknown whether the trafficking of bystander memory T cells to the tumors in absence of cognate Ag is also dependent on CXCR3. To directly test this, CRISPR-Cas9 was used to remove *cxcr3* from naïve P14 CD8 T cells (**Figure 5A**) which were then adoptively co-transferred into mice along with ATTO-only CRISPR-Cas9 (WT) controls (**Figure 5B**). The recipient mice were infected with LCMV_Arm_ (**Figure S5A**). After the *cxcr3* WT and KO populations differentiated into memory CD8 T cells (~60d P.I.), *cxcr3* knockout was confirmed (**Figure 5C**), mice were inoculated subcutaneously with a parental line of B16.F10 tumors, which did not express the GP33 epitope. Twenty-one days post-tumor inoculation, tissues were collected as in **Figure 1** and analyzed for donor populations. CXCR3 expression remained at high levels on greater than 90% of the WT cells isolated from the spleen and lymph nodes but was downregulated in the TILs (**Figure 5D**). As expected, expression of CXCR3 was absent on the vast majority (>90-85%) of *cxcr3* KO cells across all tissues examined (**Figure 5D**). Aside from the expression of CXCR3, the WT and KO cells isolated from the tumors and spleens were found to be similarly quiescent as determined by high expression of CD62L and CD127, and low expression of PD-1 and KLRG1 (**Figure S5B**). In addition, *cxcr3* KO cells largely retained the functional ability to express IFN-γ and TNFα following *ex vivo* restimulation with αCD3/αCD28, albeit to slightly lower levels than WT cells as reported previously (45–48) (**Figure S5C**). Interestingly, despite the lack of an antigenic target on the tumors for both the WT and *cxcr3* KO cells, a clear dissimilarity was observed in the anatomical distribution of the two populations. Although roughly equal numbers of the WT and *cxcr3* KO cells were transferred prior to tumor inoculation (46.6% *cxcr3* KO and 51.4% WT) (**Figure 5B**), of the donor cells, a skewed ratio of *cxcr3* KO : WT (~70% *cxcr3* KO and ~30% WT) cells was detected in the spleens, and lymph nodes of mice following tumor inoculation (**Figure 5D**). However, of the TILs, the frequency of donor cells that were WT increased to an average of 45% (**Figure 5D**). Furthermore, in the context of the total number of respective donor cells recovered from all analyzed tissues, a significantly greater proportion of the WT donor cells localized to the tumors compared to the *cxcr3* KO cells (as determined by the observed change in ratio of *cxcr3* KO donor cells to total donor cells) (**Figure 5E**). These findings demonstrate that similar to CXCR3-dependent trafficking of Ag-specific T cells to solid tumors (25, 26), CXCR3 plays a major role in the antigen-independent migration of bystander memory CD8 T cells to solid tumors.

### Bystander CD8 T Cells Are Functional in a CAR T Therapy Mouse Model

**Figure 6:**
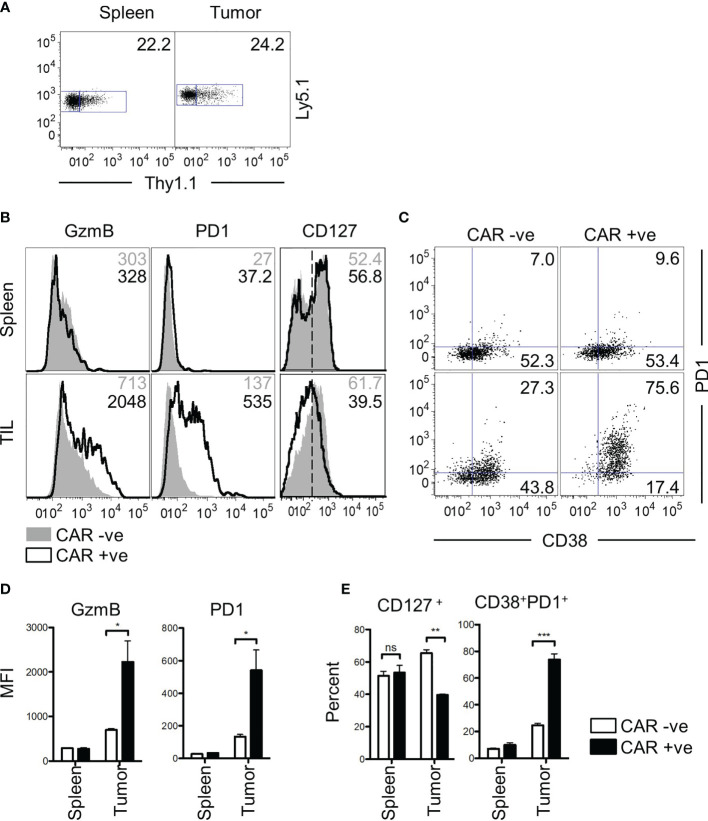
Bystander CD8 T cells are functional in a CAR T therapy mouse model. **(A)** CD8 T cells transduced with a CD19 CAR and Thy1.1 transduction marker are observed in both spleen and tumor of mice at day 25 post transfer. Donor cells express Ly5.1 and CAR transduced cells express Thy1.1. **(B)** Phenotypic markers GzmB, PD-1, CD127, and **(C)** CD127 vs CD38 were assessed by flow cytometry. Values for GzmB and PD-1 show MFI, whereas the numbers for CD127 and CD38 v PD-1 show percent gated positive or double positive. GzmB and PD1 markers are graphed below in **(D)** CD127+ and CD38+PD1+ are graphed in **(E)** All data is representative of two independent repeats with N=3 mice per group. ns, non-significant. *p < 0.05, **p < 0.01, ***p < 0.001 as determined by paired t-test.

Finally, we asked whether the characteristics of bystander memory CD8 T cells were translatable to a CAR T cell therapy model. To test this, we retrovirally transduced P14 CD8 T cells with an anti-CD19 CAR construct and adoptively transferred a mixed population of transduced (CAR^+^) and non-transduced (CAR^-^) T cells into naïve C57Bl/6 mice (**Figure S6A**). The mice were then infected with LCMV_Arm_ to expand both populations of cells using the TCR. Having established the localization and functional competence of tumor non-reactive bystander memory cells in two distinct solid tumor-types, we next sought to determine if CAR-T cells that are nonreactive to tumor antigens also localize to tumors and retain functionality. Since *in vivo* expansion in response to cognate antigen on tumors is essential for CAR-T cell detection, we engaged the strategy of LCMV infection to expand CAR T cells generated using P14 T cells through the H2D^b^ : GP33-specific TCR six days after infection, mice were inoculated subcutaneously with MC38 tumors expressing truncated hCD19 (hCD19t) antigen as a model tumor-associated antigen (**Figure S6A**). The CAR^+^ and CAR^-^ T cells were detectable in roughly similar proportions in both the spleen and tumor sites 25 days after transfer (**Figure 6A**). Phenotypic analysis of the CAR^+^ and CAR^-^ donors showed no significant differences in the cells isolated from the spleen (**Figure 6B, D**). Similar to the TCR-based models of the bystander donor population that infiltrated the tumors, the CAR^-^ T cells, which acted as bystanders in this model, did not express effector protein GzmB, or exhaustion markers PD-1 and CD38 (**Figures 6B–E** **)**. In contrast, majority of the CAR^+^ T cells isolated from the tumors exhibited increased expression of GzmB, PD-1, and CD38 compared to the CAR^+^ cells from the spleen (**Figures 6B–E** **)**. Furthermore, compared to the CAR^+^ cells, the bystander CAR^-^ TILs exhibited memory phenotype, based on increased expression of CD127 (**Figure 6B, D**), similar to the data in **Figure 1**. Finally, compared to the CAR^+^ cells, a greater frequency of CAR^-^ T cells retained polyfunctionality following residency in the TME as determined by expression of both IFN-γ and TNFα following *in vitro* restimulation (**Figure S6B**). Tumor cell expression of hCD19t was confirmed at the experimental end-point, thus indicating that the CAR T cells in the tumors had persisted under chronic antigenic conditions (**Figure S6D**). Collectively, these data mirror the results from the bystander memory cells in the TCR-based models, and further support that chronic antigenic signaling through CARs in the TME is the central driver of CAR T cell exhaustion in solid tumors. Importantly, these data also provide evidence that bystander memory T cells display a similar phenotype, and functionality in both TCR and CAR-based models.

## Discussion

The limited success of current adoptive T cell therapies against solid tumors has widely been attributed to the milieu of immunosuppressive factors present within the TME (4–7). While these variables almost certainly contribute to tumor-specific T cell exhaustion and subsequent loss of function, the results from this study suggest that extended exposure to these extrinsic factors alone are not sufficient to drive terminal exhaustion in T cells. In the context of TCR and CAR-based models of T cell immunity against solid tumors, here we show that in the absence of antigenic signaling, bystander memory T cells retain a quiescent phenotype and functional potency in both immunogenically hot MC38 carcinoma, and cold B16.F10 melanoma tumors (49–51). These findings are consistent with similar reports in distinct murine and human tumors as well (19, 20, 24, 52). The differences in functionality observed between the bystander memory cells recovered from spleens, and TILs, or between TILs recovered from MC38 and B16.F10 tumors were not found to be statistically significant (**Figure 3B**). However, the differences observed were consistent within experimental groups and raise the possibility that tumor-specific factors such as the composition of cytokines (immunosuppressive *vs* pro-inflammatory), costimulatory or inhibitory molecule signaling on tumor or immune cells, and access to nutrients and metabolites may impact bystander memory functionality. The antigenic encounter history of memory T cells is also likely to impact the functionality of bystander memory cells in the TME. For example, primary, secondary, tertiary and quaternary bystander memory T cells are expected to show progressively higher functionality in the TME, as suggested by recent report of increased responsiveness to inflammatory signaling and tumor control by memory cells that have encountered multiple rounds of antigenic restimulation (53). In rigorous functional tests, our studies show that primary bystander memory CD8 T cells retained robust polyfunctionality when restimulated with cognate antigen even *in situ* in the immunosuppressive tumor microenvironment. Thus, our findings are consistent with those of Rosato et al. who demonstrated that the activation of bystander memory T cells *via* antigenic signaling can augment the anti-tumor response (19). Tumor-derived bystander memory cells further exhibited robust recall expansion potential *in vivo* and were capable of undergoing potent expansion and effector differentiation upon rechallenge, thus further supporting the notion that they did not adopt a terminally differentiated state. Due to the largely isolated nature of solid tumor Ag to the tumors, our T cell transplant results demonstrate that functional T cells can persist and function as long-lived memory T cells in extra-tumoral sites reinforce the hypothesis that chronic antigenic signaling is the driving force behind T cell exhaustion and subsequent dysfunction in the TME.

CXCR3 has been identified as a key chemokine receptor for efficient localization of CD8 T cells to solid tumors (54–56). Loss of CXCR3 or its ligands, CXCL9 or CXCL10, has been shown to disrupt the migration of adoptively transferred T cells to solid tumors resulting in impaired anti-tumor responses (25, 26, 57). That the bystander memory T cells showed a dependency on CXCR3 for effective tumor migration is consistent with these results and reinforces that CXCR3-mediated T cell trafficking to tumors can occur in an antigen-independent manner (46). As CXCR3 is upregulated following CD8 T cell activation and is maintained on effector and memory cells independently of continued antigenic stimulation (47), the superior trafficking of the bystander memory T cells compared to the naïve tumor-specific T cells in this study was likely attributed to their previously-activated state.

Although CXCR3 appeared to play a significant role in T cell trafficking to solid tumors, a notable number of *cxcr3* KO cells were present in the TILs. Interpretation of these results is complicated due to both antigen specific and bystander memory cell downregulation of CXCR3 expression within the TME. The loss of CXCR3 expression has been observed in multiple solid tumor types and is likely attributed to cell-extrinsic variables within the TME such as inhibitory receptor signaling and TGF-β secreted by tumor cells (58). In light of these observations, it is possible that the CXCR3 KO donor TILs stemmed from the minority population that did not successfully ablate CXCR3 expression, but downregulated CXCR3 expression after reaching the tumor sites. It is also possible that the trafficking of *cxcr3* KO T cells to the tumors was facilitated by other redundant chemokines that assume a dominant role in the absence of CXCR3. A recent study showed that CXCR3 was critical for responsiveness to checkpoint blockade immunotherapy by increasing T cell proximity to intratumoral CD103^+^ DCs in the TME (45). Consistent with this report, our studies suggest that CXCR3 overexpression may be exploited in adoptive T cell immunotherapy to drive the trafficking of tumor-reactive T cells to tumor-sites and synergize with PD-1 checkpoint blockade immunotherapy. The multi-faceted role of CXCR3 expression in cancer biology warrants further investigation, especially in the context of diverse solid tumor-types and adoptive TCR and CAR T cell therapy.

Furthering the observations that bystander T cells are present in solid tumors, our studies provide deeper insight into the phenotype and functional capabilities of memory bystander T cells within the TME. Our findings that tumor infiltrating bystander CD8 T cells do not exhibit hallmarks of exhaustion, such as sustained inhibitory receptor expression, loss of cytokine production (4) and terminal differentiation (59), bear implications for future exploitation of bystander tumor-resident memory T cells for indirect augmentation of tumor-reactive T cells during checkpoint blockade immunotherapy as suggested by Rosato et al. (19),. Importantly, our results directly show that without chronic antigenic signaling, T cells within the TME retain functionality and memory potential. As strategies emerge to control the expression of CARs (29, 34) as well as modulate TCR/CAR signaling (28, 60, 61), our findings reinforce the potential for strategies to mitigate T cell exhaustion in the TME by regulating TCR/CAR expression and/or signaling, thereby augmenting the therapeutic efficacy of adoptive T cell transfers against solid tumors.

## Data Availability Statement

The original contributions presented in the study are included in the article/**Supplementary Material**. Further inquiries can be directed to the corresponding author.

## Ethics Statement

The animal study was reviewed and approved by IACUC, Seattle Children’s Research institute.

## Author Contributions

PS and SJR conducted the experiments, analyzed the data, interpreted the results, and prepared the manuscript. VK and SS conceptualized the project, designed the experiments, supervised the work, analyzed the data, interpreted the results, and prepared the manuscript. All authors contributed to the article and approved the submitted version.

## Funding

This work was supported by research funding from the American Cancer Society to SS, the Pediatric Cancer Research Foundation to SS, the Rachel Lynn Henley Foundation to VK, the National Institutes of Health (AI132819 to SS and AI103748 to SS; 5P30CA015704 and AI154363 to VK), and seed funds from the Seattle Children’s Research Institute to SS and VK.

## Conflict of Interest

The authors declare that the research was conducted in the absence of any commercial or financial relationships that could be construed as a potential conflict of interest.

## Publisher’s Note

All claims expressed in this article are solely those of the authors and do not necessarily represent those of their affiliated organizations, or those of the publisher, the editors and the reviewers. Any product that may be evaluated in this article, or claim that may be made by its manufacturer, is not guaranteed or endorsed by the publisher.
