# Supplementary material for: Solid Tumor Microenvironment Can Harbor and Support Functional Properties of Memory T Cells
Source: Front Immunol. 2021 Nov 11;12:706150. doi: 10.3389/fimmu.2021.706150 (PMC8632651; doi:10.3389/fimmu.2021.706150)
Supplement: Supplementary file 1 [file DataSheet_1.pdf]

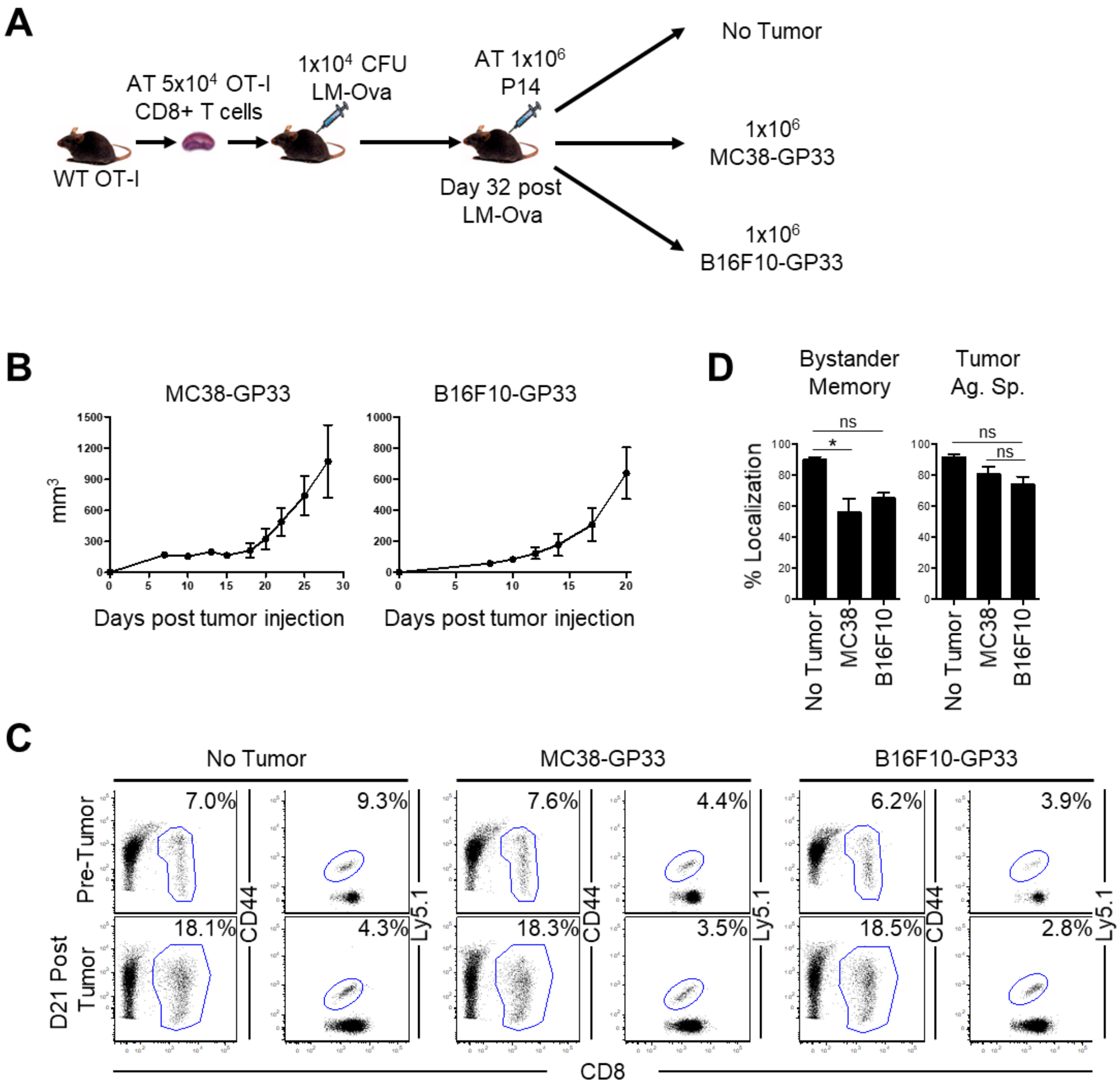

Supplementary Figure 1. Bystander Memory T Cells infiltrate into established solid tumors. **A.** Female OTI spleen was collected. 50,000 OT-I specific CD8 T cells were adoptively transferred to B6 mice, which were subsequently infected with 1000 CFU LM-Ova. After memory differentiation (> day 30), $1x10^6$ naïve P14 CD8 T cells were adoptively transferred into the OT-I memory mice. $1x10^6$ tumor cells of either MC38-GP33 or B16.F10-GP33 were injected subcutaneously. Mice were sacrificed once tumor size approached $1000mm^3$ and tissues were collected. **B**. Tumor volume was monitored by caliper measurements every 2-3 days and calculated as volume=(length*width^2)/2. **C**. PBMC from pre-tumor and from day 21 post-tumor injection were analyzed for bystander memory cell ($CD8^+$, $Ly5.1^+$) frequency in mice which received no tumor, MC38-GP33, or B16.F10-GP33 tumors. **D**. Percent localization of bystander memory and tumor antigen specific CD8 T cells in the spleen from mice with no tumor, MC38-GP33, or B16.F10-GP33 tumors. * $p<0.05$ as determined by ANOVA with Dunnett's Multiple Comparison.

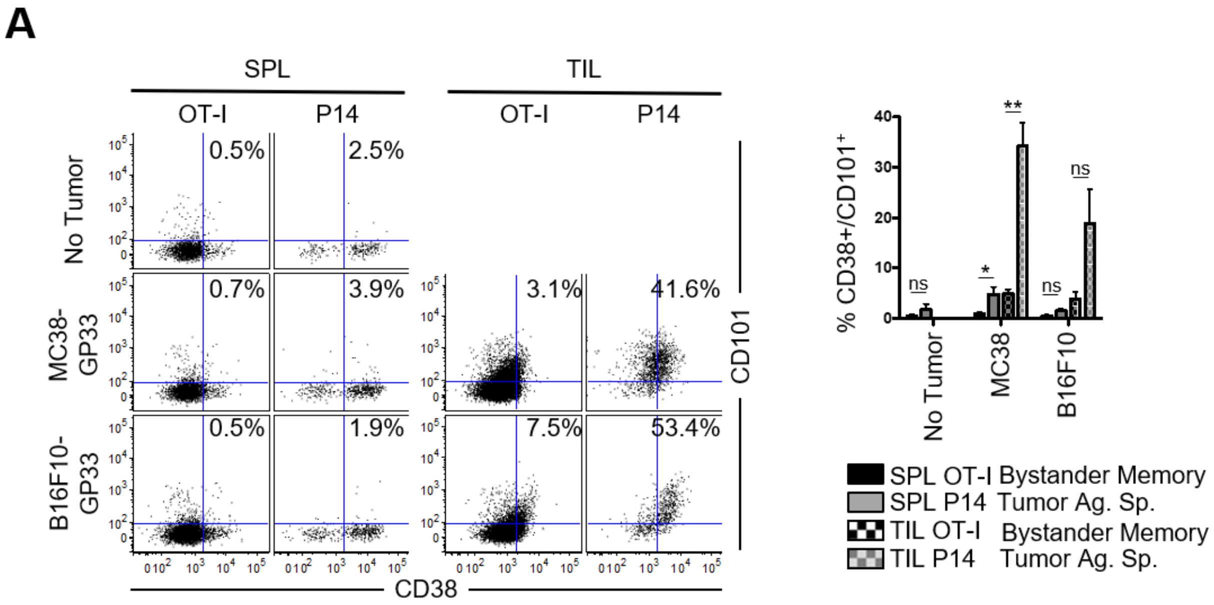

Supplementary Figure 2. Phenotype of bystander memory T cells in tumor microenvironment. A. Representative CD101 v CD38 dot plots from spleen and TILs of memory OT-I mice with no tumor, MC38-GP33 tumor, or B16.F10-GP33 tumor. Percentage of CD38 positive, CD101 positive cells are shown in black. Bar charts show the percent of CD38/CD101 double positive cells and the percent of total CD38 positive cells. * $p<0.05$. ** $p<0.01$ as determined by paired t-test.

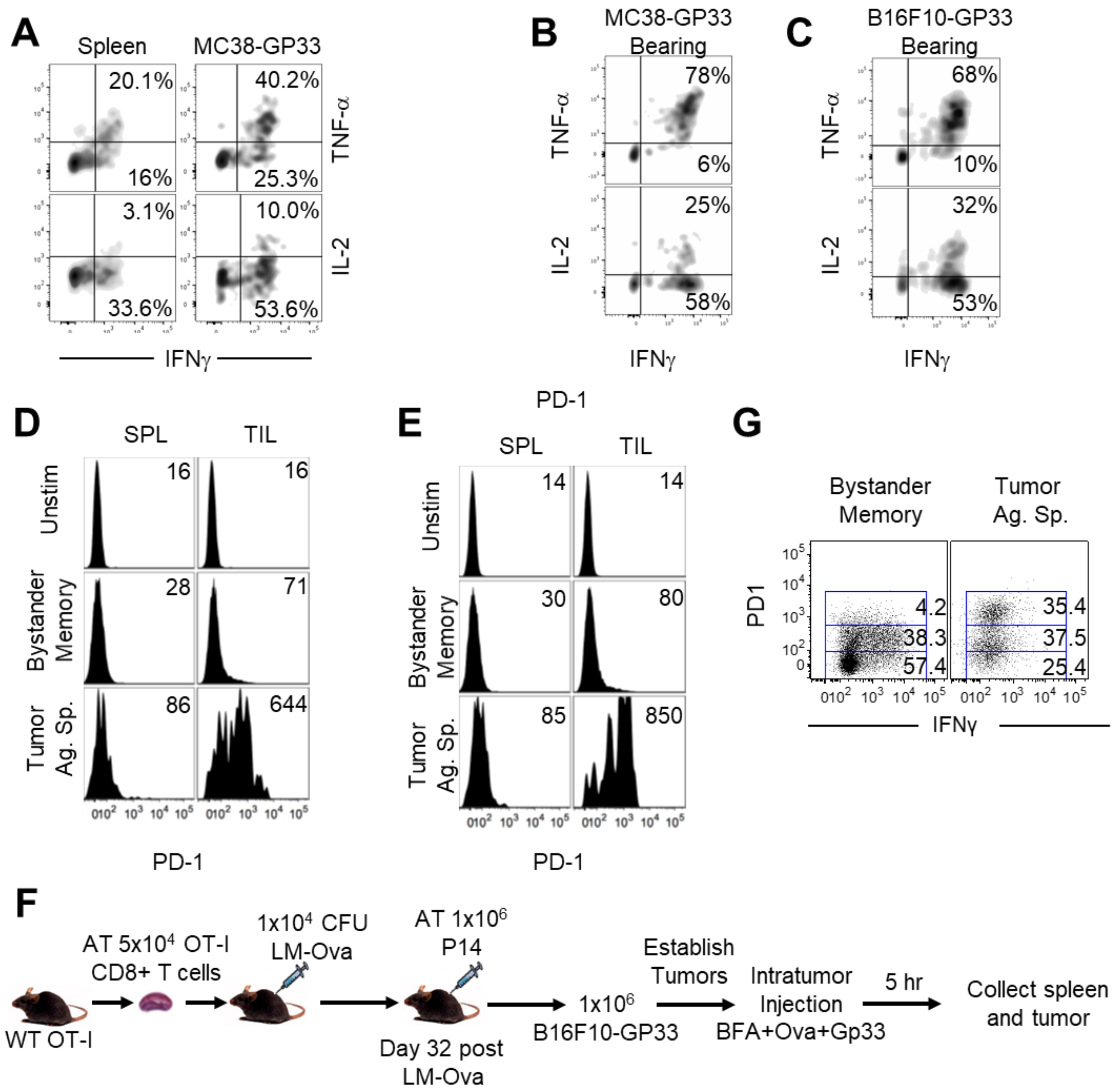

Supplementary Figure 3. Polyfunctionality of bystander memory T cells in tumor microenvironment **A**. Memory bystander cells from the spleen or TILs were stimulated with αCD3/αCD28 for 5 hours in the presence of BFA and cytokine production was assessed. FACS plots show % of IFN-γ/TNF-α double positive or % IFN-γ/IL-2 double positive. **B, C**. Tumor-Ag. Sp. donor cells from the spleen of either MC38-GP33 tumor-bearing mice (**B**) or B16.F10-GP33 tumor-bearing mice (**C**) were stimulated with GP33 peptide for 5 hours in the presence of BFA, then cytokines production was assessed. FACS plots show % of IFN-γ/TNF-α double positive or % IFN-γ/IL-2 double positive of each population. **D, E**. PD-1 histograms from unstimulated CD8 T cell population (Unstim), Bystander memory stimulated with αCD3/αCD28, and Tumor Ag. Sp. stimulated with αCD3/αCD28 in both spleen and tumors of MC38-GP33 (**D**) or B16.F10-GP33 (**E**) tumor bearing mice. Mean fluorescence intensity is shown in the upper right corner. **F**. Established B16.F10-GP33 tumors were injected intratumorally with 30 ul PBS containing Ova peptide, GP33 peptide, and BFA. Five hours later, the spleen and tumors were collected, and cells were assessed for cytokine production. **G**. FACS plot of IFN-γ against PD-1 from *in situ* activation of donor CD8 T cells gated on low, intermediate, and high PD-1 expression. Percent of cells in each gate are shown.

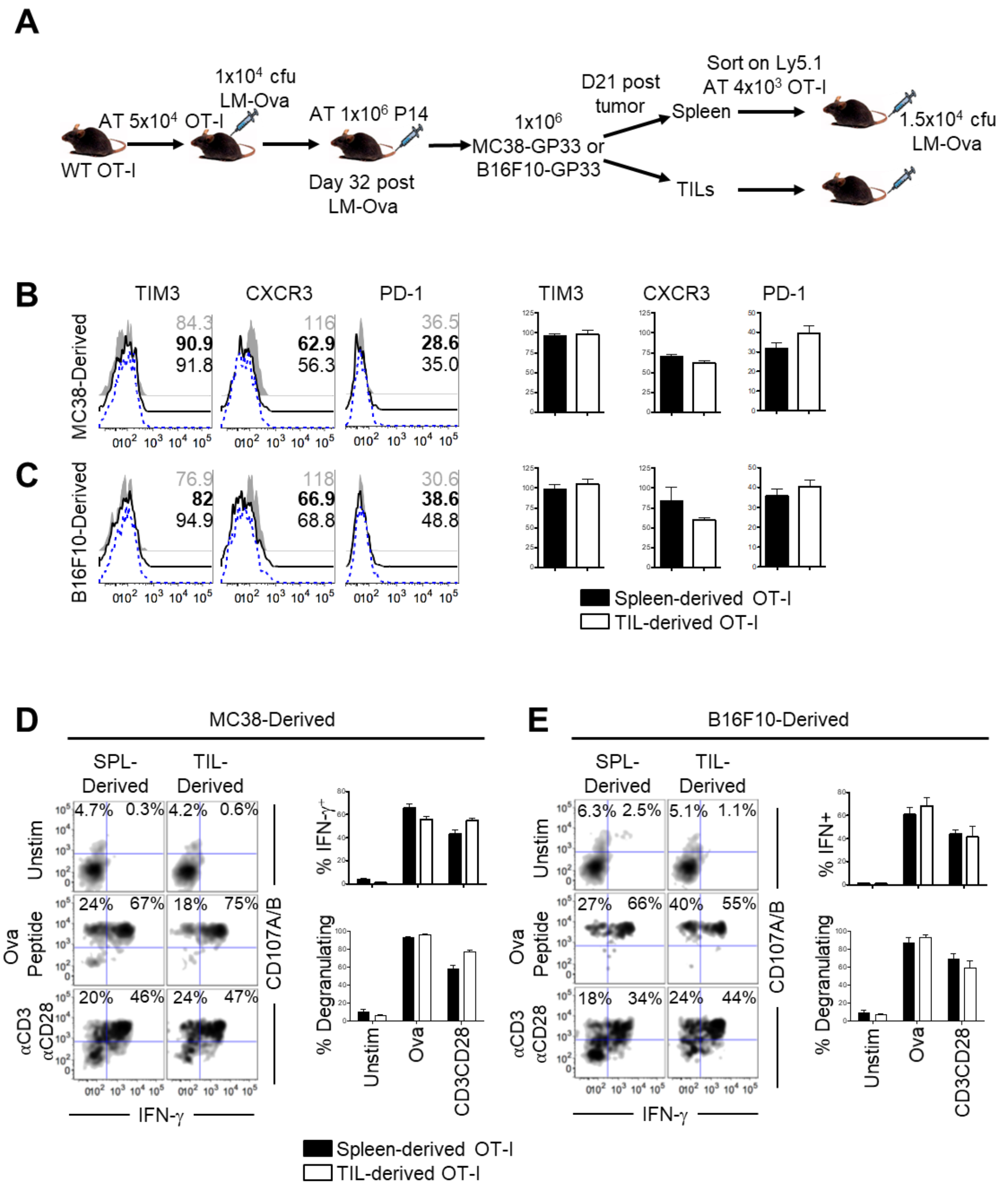

Supplementary Figure 4. Bystander memory CD8 T cells from the tumor microenvironment retain their recall ability. A. Schematic of experimental outline. Bystander memory CD8 T cells were isolated from TILs or spleens of mice bearing B16.F10-GP33 tumors and were sorted by FACS. 4000 OT-I bystander memory cells were adoptively transferred in naïve B6 mice and subsequently infected with 15k CFU of LM-Ova. **B, C**. Spleens samples of mice that received donors originating from MC38-GP33 bearing mice (**B**) or from B16.F10-GP33 (**C**) were stained for phenotypic markers. Histograms depict the mean fluorescence intensity (MFI) of the given marker. Quantification of each marker is shown to the right. T-tests were run to compare the SPL-derived donors to the TIL-derived donors with no significant differences found. **D, E**. Splenocytes from were stimulated with Ova-peptide or αCD3/αCD28 for 5 hours in the presence of BFA, monensin, and CD107A/B antibody. IFN production was assessed by intracellular staining and flow cytometry. Representative flow plots are shown with the percentage of degranulating (CD107A/B$^+$), IFN-γ$^+$ cells. The percentage of IFN-γ$^+$ and degranulating donor cells are shown in bar charts to the right. Statistical significance was determined by unpaired t-test without any differences identified.

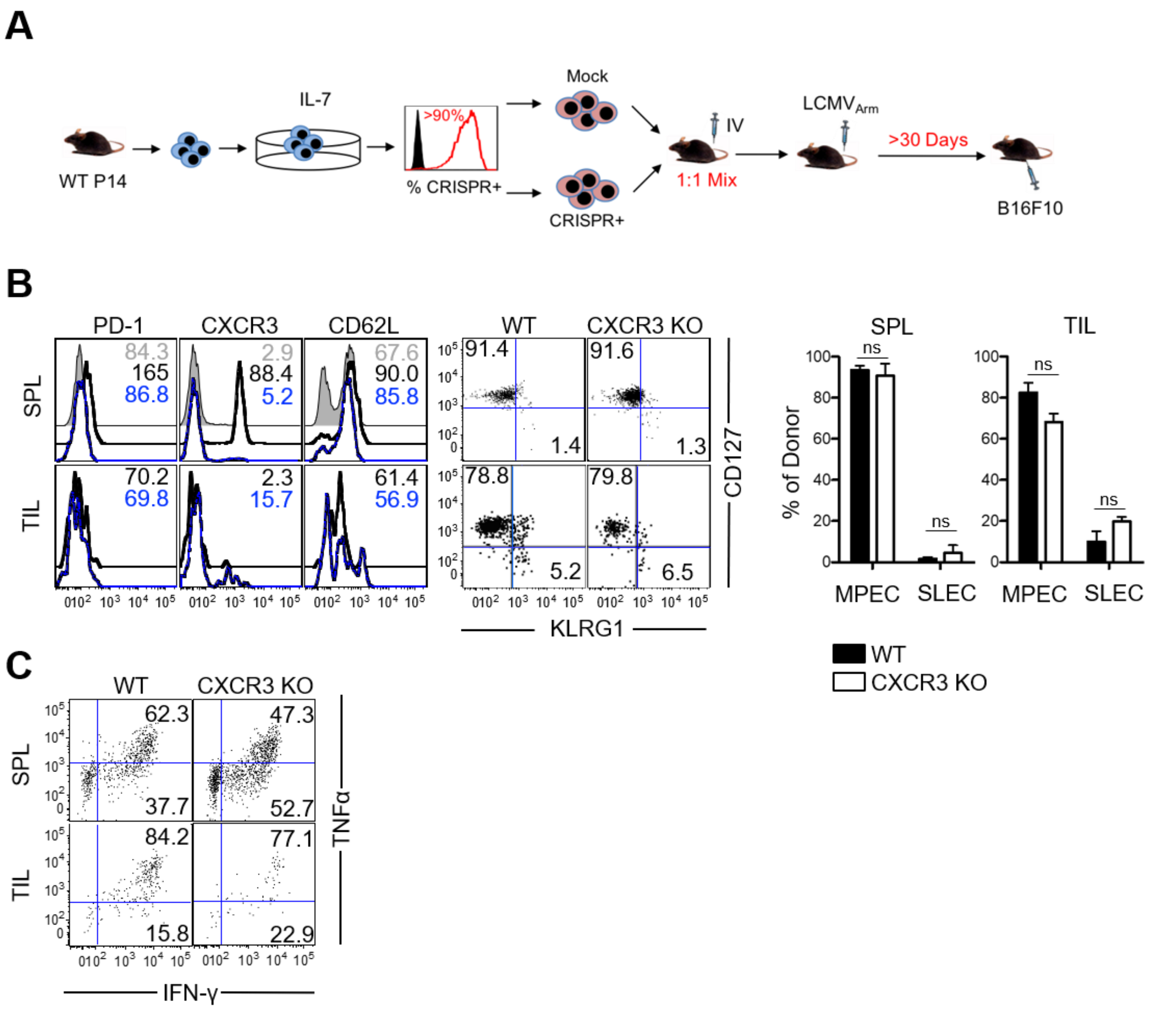

Supplementary Figure 5. CXCR3 is necessary for proper localization of bystander memory cells to the tumor. A. Schematic of experimental set up. WT P14 from Thy1.1/1.1 and Thy1.1/1.2 were incubated with IL-7 overnight. CRISPR/Cas9 targeting CXCR3 was introduced by electroporation. The Cas9 uptake was validated by ATTO550 signal, determined by flow cytometry. Cells were then mixed 50:50 and $1x10^5$ total cells were adoptively transferred into B6 mice. Mice were infected with LCMV$_{Arm}$ and the P14 cells were allowed to progress to memory T cells. $1x10^5$ WT B16.F10 tumors were injected subcutaneously. Twelve days post tumor injection, the tissues were collected and analyzed. **B**. Wild Type and CXCR3 KO Splenocytes and TILs were analyzed by flow cytometry for surface markers PD-1, CXCR3, and CD62L. The memory status of the cells was assessed by CD127 and KLRG1 expression. The percent of MPEC and SLEC of each donor population is graphed to the right. **C**. Functionality of the wild type and CXCR3 KO cells was assessed by *ex vivo* cytokine stimulation using αCD3/αCD28 stimulation. IFN-γ and TNFα are plotted to demonstrate functionality in both sets of cells. The percentage of $IFN\gamma^+TNF\alpha^+$ or $IFN\gamma^+TNF\alpha^-$ of total $IFN\gamma^+$ cells is shown in the corresponding quadrants. Statistical significance was determined by unpaired t-test without any differences identified.

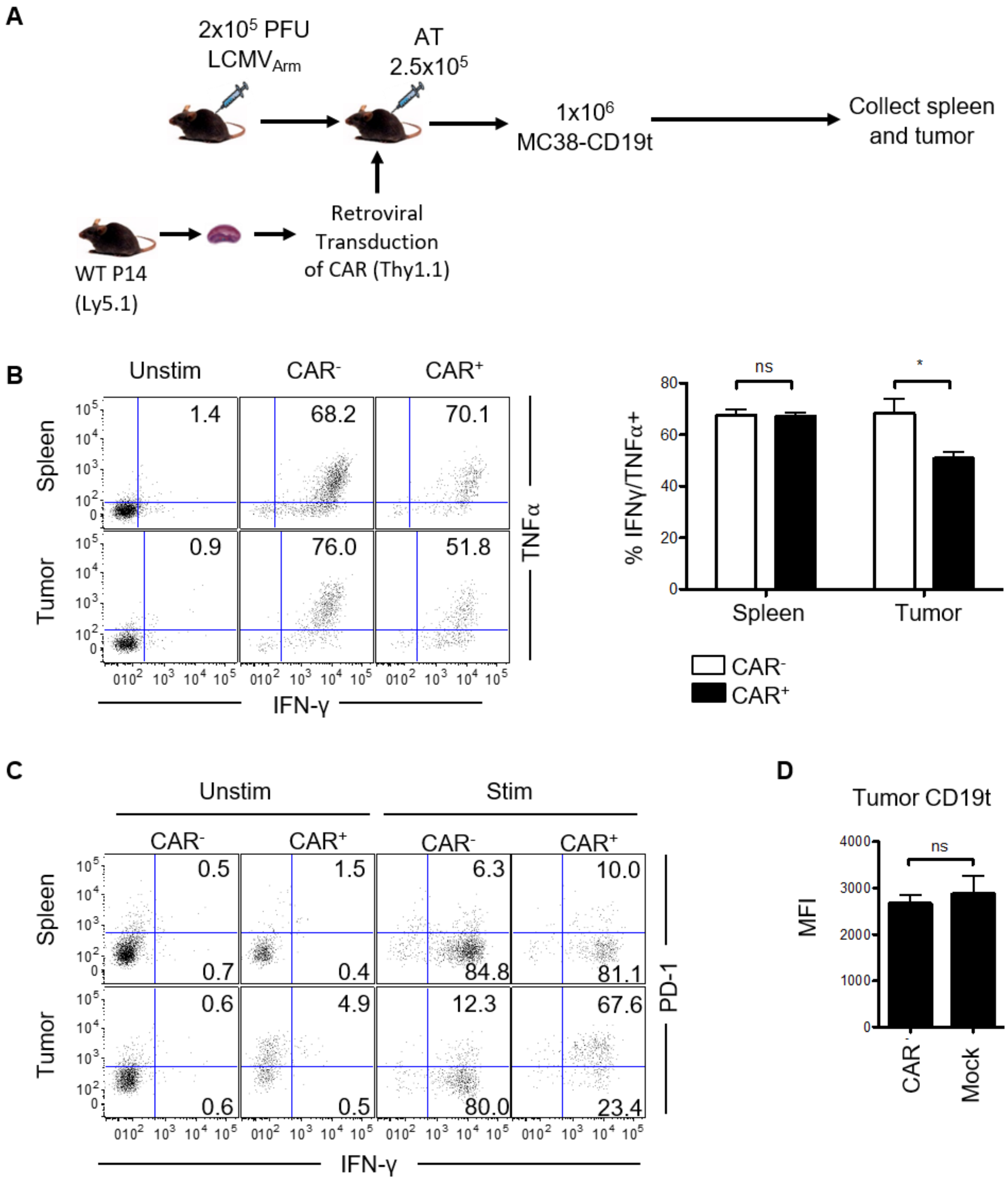

Supplementary Figure 6. Bystander CD8 T cells are functional in a CAR T therapy mouse model. A. Experimental setup. WT P14 mice expressing the cogenetic marker Ly5.1 were activated *in vitro* for 24 hours, then transduced retrovirally to express CD19 CAR and Thy1.1. 2.5x10^5 cells were adoptively transferred into day 1 $LCMV_{Arm}$ infected B6 mice. MC38-CD19t tumors were injected subcutaneously into the flank of the mice 6 days post transfer. Spleen and tumors were collected 25 days post adoptive transfer. **B.** Tumor or spleen samples were stimulated *ex vivo* in the presence of BFA for 5 hours. Cytokine production was assessed by IFN-γ and TNFα expression. Percent of double positive producers is plotted to the right. **C.** PD-1 expression was plotted against IFN-γ for unstimulated and stimulated spleen and tumor samples. Numbers show the percent of each quadrant. **D.** CD19t expression in tumor cells was assessed to ensure that CAR target antigen was not negatively selected against during tumor growth. * $p<0.05$ as determined by paired t-test.
